# Clinical benefit of high-volume hemofiltration in severe burn injury: is it removing bad humors or actually avoiding hypervolemia?

**DOI:** 10.1186/s13054-018-2230-7

**Published:** 2018-11-01

**Authors:** Patrick M. Honore, David De Bels, Thierry Preseau, Sebastien Redant, Rachid Attou, Andrea Gallerani, Herbert D. Spapen

**Affiliations:** 1 ICU Department, Centre Hospitalier Universitaire Brugmann—Brugmann University Hospital, Place Van Gehuchtenplein,4, 1020 Brussels, Belgium; 2 Emergency Department, Centre Hospitalier Universitaire Brugmann, Brussels, Belgium; 3 Universitair Ziekenhuis Brussel, VUB University, Brussels, Belgium

Recently, You et al. [1] reported that early application of high-volume hemofiltration (HVHF) reduced the incidence of sepsis, septic shock, and organ failure in patients with burns ≥ 50% total burn surface area (TBSA) and improved survival of patients with burns ≥ 80% TBSA. The benefit of HVHF was attributed to hemofiltration/adsorption of proinflammatory cytokines and other sepsis-related mediators and recovery of the patients’ immune status [1]. However, HVHF as adjuvant therapy for sepsis has previously been shown to have no significant impact on hemodynamics, short-term morbidity and mortality, and cytokine clearance [2, 3]. The results from You et al. are more remarkable because HVHF was performed with a less adsorptive dialysis membrane and a relatively low prescribed effluent rate [2, 3]. Moreover, 70–100% of the replacement solution was administered in predilution, which makes convective mediator removal less effective as compared to full postdilution [2, 3].

Cytokine levels fell most during the 3-day HVHF treatment. Thereafter, levels followed a rather “flat” course in both treatment groups without surges reflecting the occurrence of sepsis or septic shock. No data are available regarding the origin (pulmonary, catheter, etc.) or type (Gram-negative or Gram-positive, fungal) of sepsis, all of which may determine cytokine and mediator fluxes. It is also questionable that a lower procalcitonin (PCT) level in the HVHF group represents less inflammation. PCT (molecular weight 13 kDa) indeed is easily removed by any form of convective continuous renal replacement therapy, thereby losing sensitivity to detect or assess inflammatory processes [4].

The most striking finding is the rapid reversal of shock as mirrored by less vasopressor dependency in the HVHF-treated patients. Liberating patients from vasopressor treatment is of utmost importance in burn patients, not only as an indicator of hemodynamic stabilization but particularly to avoid graft ischemia, rejection, and surinfection [3]. The authors associate this “acute” effect with HVHF-induced immune-inflammatory modulation [1]. Of note is that no information is provided on fluid administration and evolution of fluid balances. Patients with severe burn injury receive massive fluid loads during the first days of treatment. Early de-resuscitation (i.e., evacuation of excess fluid aiming at zero fluid balance around day 3 of treatment) is a crucial factor to improve morbidity and perhaps mortality in critically ill and injured patients [5]. HVHF may thus have acted as an excellent method to rapidly reach de-resuscitation targets.

## Abbreviations

HVHF: High-volume hemofiltration
PCT: Procalcitonin
TBSA: Total burn surface area
